# Migration of Fish

**Published:** 1883-01

**Authors:** 


### MIGRATION OF FISH.

Dr. Keller, in a communication sent to the Swiss Geographical Society, from Suez, gives some interesting points on this subject. In the twelve years that have elapsed since the opening of the Suez Canal, the interchange of animal life between the Mediterranean Sea and the Indian Ocean has not reached the dimensions at first anticipated, still a number of smaller fish have found their way from the Mediterranean to the Red Sea. A greater desire to travel in this direction than in the opposite one seems to prevail. A very interesting fact has, however, been established, namely, that the real pearl oysters are traveling through the canal, not a few straggling outposts, but large trains moving regularly along. As they have not yet reached the Timsah lake, it will be one or two decades before they will be established in the Mediterranean.

